# Human pluripotent stem cells on artificial microenvironments: a high content perspective

**DOI:** 10.3389/fphar.2014.00150

**Published:** 2014-07-02

**Authors:** Priyalakshmi Viswanathan, Terri Gaskell, Nathalie Moens, Oliver J. Culley, Darrick Hansen, Mia K. R. Gervasio, Yee J. Yeap, Davide Danovi

**Affiliations:** ^1^HipSci Cell Phenotyping, Centre for Stem Cells and Regenerative Medicine, Guy’s Hospital, King’s College LondonLondon, UK; ^2^Cell Therapy Catapult, Guy’s HospitalLondon, UK

**Keywords:** phenotyping, pluripotent stem cells, microenvironment, high content, single cell

## Abstract

Self-renewing stem cell populations are increasingly considered as resources for cell therapy and tools for drug discovery. Human pluripotent stem (hPS) cells in particular offer a virtually unlimited reservoir of homogeneous cells and can be differentiated toward diverse lineages. Many diseases show impairment in self-renewal or differentiation, abnormal lineage choice or other aberrant cell behavior in response to chemical or physical cues. To investigate these responses, there is a growing interest in the development of specific assays using hPS cells, artificial microenvironments and high content analysis. Several hurdles need to be overcome that can be grouped into three areas: (i) availability of robust, homogeneous, and consistent cell populations as a starting point; (ii) appropriate understanding and use of chemical and physical microenvironments; (iii) development of assays that dissect the complexity of cell populations in tissues while mirroring specific aspects of their behavior. Here we review recent progress in the culture of hPS cells and we detail the importance of the environment surrounding the cells with a focus on synthetic material and suitable high content analysis approaches. The technologies described, if properly combined, have the potential to create a paradigm shift in the way diseases are modeled and drug discovery is performed.

## PLURIPOTENT STEM CELLS AND THEIR CULTURE

### HUMAN PLURIPOTENT STEM (hPS) CELLS ARE DERIVED FROM EMBRYOS OR THROUGH REPROGRAMMING

Stem cells are defined as cells capable of self-renewal, the capacity to generate identical copies of themselves, and differentiation, the ability to provide cells performing a specific biological function (Smith, 2006).The capacity to differentiate into all lineages sufficient to form an entire organism, and not necessarily extra embryonic tissues, is defined as pluripotency. This property can be demonstrated by showing differentiation into cells from the three germ layers (endoderm, mesoderm, and ectoderm). With no test available for germline transmission of human cells, pluripotency can be demonstrated in immune-deficient mice by the ability to form teratomas. *In vitro,* a number of molecular markers are used as a surrogate for pluripotency; some of these carry functional significance, such as Oct4 and Nanog, whereas others are considered mostly descriptive, such as stage-specific embryonic antigen (SSEA)-4 and Trafalgar antigen TRA-1-60.

Using diverse culture methods, several types of cells have been characterized which broadly fit many aspects of the definition of pluripotency. hPS cells are derived from transient populations of cells isolated from the embryo such as human embryonic stem (hES) cells or are artificially reprogrammed from somatic cells such as human induced pluripotent stem (hiPS) cells. In mice, several populations of pluripotent cells can be derived from pre- and post-implantation embryos. The earlier or naïve cells are the originally described mouse embryonic stem (mES) cells whereas the post implantation cells (epiblast stem cells or epistem cells) represent a later stage of development and are thought to be “primed” to differentiate with potential lineage bias (Nichols and Smith, 2009)

In human, however, hES cells resemble more closely the mouse epistem cells (Tesar et al., 2007; Greber et al., 2010; Ware et al., 2014). Moreover, whilst hES cells fall into the “primed” category with hiPS cells, the characteristics can vary depending on somatic source, reprogramming method and culture system. In recent times, many researchers have attempted to define conditions under which naïve hPS cells can be derived and maintained (Gafni et al., 2013). Importantly, in the human blastocysts there are several different cell populations that can give rise to pluripotent stem cells when explanted and cultured (Niakan and Eggan, 2013). The extensive crosstalk between stem cells and their niche encompasses neighboring cells, soluble cues, and extracellular matrix (ECM) proteins and is key to the maintenance of pluripotency. It is also likely that in the early phases, the surrounding environment plays a major role in instructing cells to enable self-organizing properties as has been reported in the mouse system (Bedzhov and Zernicka-Goetz, 2014). Unsurprisingly then, at each step of the evolution of culture systems, the emphasis has been on recapitulating the “natural” environment, or “niche” (Lutolf and Blau, 2009). Along these lines, the secretion of growth factors by stromal cells has informed the choice of factors and more recently attention has been devoted to mimic the structural and mechanical properties of the natural niche. However, although a tractable model system, cell culture is artificial by definition and it is not easy to pinpoint what the “natural” conditions are *in vivo* and should be *in vitro*.

### DEFINING THE CULTURE: FEEDERS AND MEDIA

Irrespective of the biological differences, expansion of homogeneous starting populations in self-renewing conditions is key to realizing the promise of hPS cells for screening and modeling strategies. hPS cell culture has progressed a long way from the initial derivation and expansion on mouse feeders in medium containing bovine serum (Thomson et al., 1998). Nonetheless, production of large numbers of stable, homogeneous, and undifferentiated cells in standardized protocols is still far from a trivial matter. Mirroring progress obtained a decade in advance with mES cell culture, culture of hPS cells has evolved substantially. In fact it moved from mouse feeders to defined feeder-free systems taking in human feeders, conditioned medium, and complex substrates along the way.

There are some disadvantages associated with each of these variations. The use of feeders brings additional variability to the culture, particularly crucial if the cells are non-human. It has been shown that animal products can modify as well as contaminate hPS cells (Moore, 2006). An important factor for variability in hPS cell yield and viability is the effect of feeder cell density (Heng et al., 2004), often inconsistent across laboratories. A comparison of the literature reveals the use of a large range of seeding densities, from 20,000 to 75,000 cells/cm^2^ (Zhou et al., 2009). Human feeders although expensive, hard to maintain and equally variable have allowed the relatively early establishment of clinical grade hES cell lines (Tannenbaum et al., 2012).

Using feeder free cell culture has the advantage of removing the requirement for parallel culture and mitotic inactivation of feeder lines. Yet, it often involves conditioned medium or xenogenic complex substrates. The use of xeno-free, defined products can improve robustness and there are a number of combinations now available that do not contain animal derived components or complex additives such as sera. As implied above, selected culture systems will result in subtly different populations. Whilst still fitting the wider definition of hPS cells, these will respond in different ways to external stimuli. Therefore the lack of consensus about culture systems does pose a hurdle when comparing data between laboratories. Also, it is important to stress that established differentiation protocols will not necessarily transition seamlessly to a different culture system and can therefore represent a high barrier to progressing culture conditions even when long-term gains are significant.

Different media used for the different systems have been thoroughly and recently reviewed elsewhere (Chen et al., 2014). Here, we will briefly describe selected defined media for feeder free culture. A number of defined media for hPS cells are commercially available such as mTeSR1/2 (STEMCELL Technologies), StemPro (Invitrogen), Pluripro (Cell Guidance Systems), PluriSTEM (Millipore), Stemline (Sigma), and Nutristem (Stemgent). Most of these contain bovine serum albumin (BSA) along with a complex mixture of amino acids, trace elements, hormones, and growth factors. Human serum albumin (HSA) is present in TeSR2 whereas derived from it, the more recent Essential 8 (Invitrogen) medium does not contain HSA or BSA and can perhaps be considered a truly defined medium. Most commercially available and homemade media contain both fibroblast growth factor 2 (FGF2) and transforming growth factor β(TGFβ)/Activin A/NODAL at varying concentrations. Some of these media require higher concentrations of FGF2 to maintain the cells, further adding to the cost of culture.

### DEFINING THE PHYSICAL CONDITIONS: CELL–CELL CONTACT AND HYPOXIA

Unlike their murine equivalents, hPS cells poorly tolerate being separated to single cells and have historically therefore been propagated as clusters using mechanical or enzymatic methods or a combination of the two. Mechanical passaging methods are least favored when considering the scalability of the culture process. It is difficult to accurately determine cell-seeding densities, as hPS cells are kept in large clumps or colonies, using this technique. Consistent seeding densities are essential to reduce variability in hPS cell culture as they result in higher quality cells and more predictable yield.

Apoptosis induced by dissociation to single cells can be modulated by pharmacological inhibition of specific pathways involved in cell–cell adhesion. For example, the Ras homolog gene family member A (RhoA) acts on its downstream effector, Rho-associated protein kinase (ROCK) and ROCK inhibitors (ROCKi) can be added to the culture just at time of passage or throughout cell maintenance to counteract the stress induced by dissociation into single cells (Watanabe et al., 2007). Preparation of hPS cells as single cells, in the presence of ROCKi allows for more homogeneous populations and these are more amenable to automation. It has been suggested that enzymatic passaging or ROCKi can cause chromosomal abnormalities in hPS cells (Mitalipova et al., 2005). Despite initial discordances, a number of reports have now shown that normal karyotypes can be maintained after prolonged single cell passage demonstrating that single cell passage *per se* does not lead to chromosomal abnormalities (Mitalipova et al., 2005).

Various enzymes are currently used in hPS cell culture, including dispase II, collagenase IV, accutase, TrypLE Express (Invitrogen). Dispase and collagenase allow cells to remain as clusters, whereas accutase and TrypLE Expresss dissociate hPS cells into a single cell suspension. It should be noted, however, that these methods often still require manual removal of differentiated cells prior to enzyme addition which creates an obvious barrier to automation (discussed below). An alternative to enzymatic dissociation is the use of EDTA, which allows the dissociation of colonies to small clusters and works in conjunction with E8 medium on a defined substrate (Beers et al., 2012).

The normal atmospheric oxygen tension, at which hPS cells are generally cultured is 21%. *In vivo*, mammalian oxygen tension on the other hand ranges from 1.5 to 5.3% (Fischer and Bavister, 1993). As for other cell types (Parrinello et al., 2003) there have been attempts to evaluate the biological effect of hypoxia on hPS cells, for example through hypoxia-inducible factors (Mathieu et al., 2013). Low oxygen enhanced clonal recovery of hES cells and reduced the incidence of chromosomal aberrations without altering hES cell pluripotency marker expression (Forsyth et al., 2006).Moreover, it can improve pluripotency maintenance while reducing the incidence of chromosomal aberrations and reduce the occurrence of spontaneous differentiation (Forristal et al., 2010; Zachar et al., 2010). Despite these interesting findings, logistical problems severely limit the use of low oxygen in most laboratories and dedicated culture chambers have been proposed (e.g., biospherix).

### QUALITY BY DESIGN, SCALE-OUT, AND SCALE-UP

**FIGURE 1:**
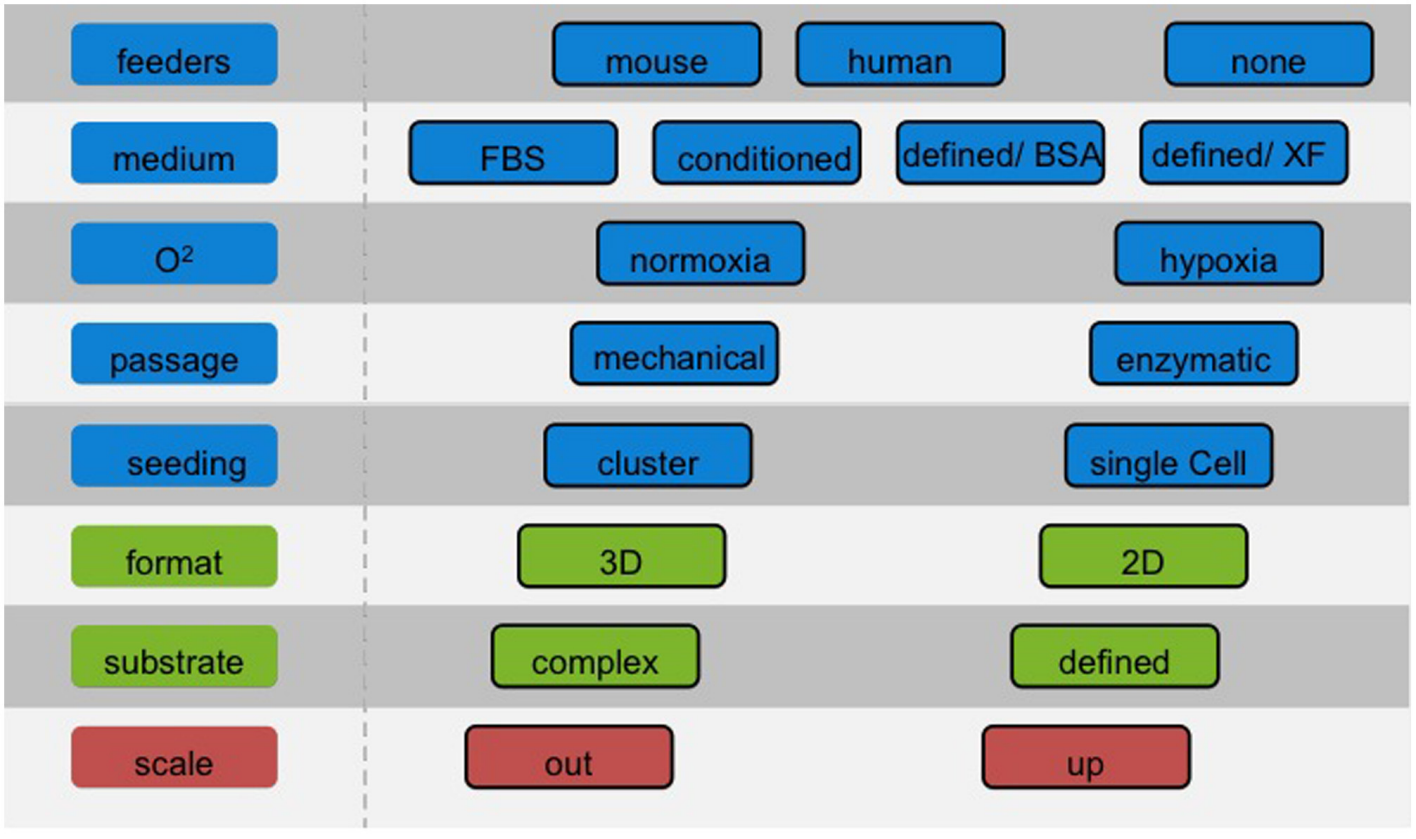
**Choosing the right cell culture conditions for hPS cells.** The number of cells required for a screening campaign is typically less substantial than for cell therapies. Nonetheless, it is of paramount importance to choose the appropriate cell culture conditions beforehand, with a clear view of the route and the end-points to achieve. Adapting culture protocols at a later stage may be problematic as cells may respond distinctly to established differentiation protocols.

The choice of the culture system has severe consequences for the potential use in different applications and a significant impact on optimization of downstream differentiation protocols. Considerations of required cell number and batch size should be addressed at an early stage to facilitate the efficient translation of protocols and avoid population drift, which will introduce variability (**Figure 1**). These problems can be minimized by establishing master- and working cell banks with limits imposed on the number of passages. Nonetheless, current bench-scale methods described above show intrinsic limitations in terms of variability and yield (Veraitch et al., 2008). Two diverse approaches can be considered to produce large numbers of cells: scale-up and scale-out.

Most 2D culture, providing manual removal of differentiated cells is not required, can be relatively easily scaled out, at least to a degree (e.g., larger or multilayer or stacked flasks) and this may be sufficient to meet certain requirements. Scaled-out systems may be especially useful for culturing multiple different cell lines at once though with high labor costs and variability. This can be addressed in part through the use of automation. Automation has been used in several steps of hPS cells expansion processes often improving consistency although not necessarily reducing process time. The first use of automation to aid hPS cells expansion was based on dissection of hPS cell colonies (Joannides et al., 2006). Subsequently studies were published in which automation was used to monitor hPS cell cultures (Narkilahti et al., 2007), to seed hPS cells and change media (Terstegge et al., 2007), to harvest hPS cells (Haupt et al., 2012) and to carry out high throughput screening as discussed below. To date, only two systems have been described that automate the full PSC expansion process. These are the CompacT SelecT (TAP Biosystems; Thomas et al., 2009) and a custom-built platform which has been tested for mES cells (Hussain et al., 2013) and is currently used to expand and differentiate hPS cells.

Scale-up methods on the other hand commonly use specialized systems such as stirred-tank reactors (STRs), spinner flasks, perfusion systems or wave bioreactors. Due to the adherent nature of hPS cell culture, cells in these systems require a surface to attach to. The use of coated beads in bioreactors can be considered as 2D culture and may not differ significantly from the output of traditional 2D culture. However, the media change dynamics are likely to have an impact on the culture conditions. STRs can contain large volumes, where culture conditions such as pH, oxygen levels, and metabolite concentrations are precisely and carefully controlled in a uniform environment with adequate nutrient levels and oxygenation (Chen et al., 2010a). To circumvent some of these problems, cells can also be microencapsulated in hydrogels in 1.1% calcium alginate capsules, which allow for the cells to remain pluripotent and proliferate for more than 8 months (Siti-Ismail et al., 2008). True 3D expansion of hPS cells in defined medium has also been reported (Zweigerdt et al., 2011) demonstrating the potential of this approach for scale-up. However, aggregated pluripotent culture pose problems and cell damage can be attributed to shear force (Serra et al., 2012). Overall, the use of scale-up systems brings considerable advantages for culture control; pH and dissolved oxygen tension can be monitored and controlled throughout the cell expansion process, which cannot be done in static culture. Perfusion systems permit the removal of waste products and addition of fresh media as required.

For feeder-free culture of hPS cells (including on microcarrier beads) the most commonly used substrates are gelatinous extracts such as Matrigel (BD Biosciences) or Geltrex (Life technologies). These are undefined extracellular matrices derived from Engelbreth–Holm–Swarm (EHS) sarcoma and are susceptible to high batch-to batch variability. Although the use of these substrates can eliminate feeders from the culture system, certain components remain unknown. More defined substrates that can support single cell passage are fibronectin, vitronectin, and laminin. Laminin-521 (Rodin et al., 2014) or laminin-511 E8 fragment (Nagakawa et al., 2014) have also been proposed. Alternative xeno-free substrates, such as CellStart (Invitrogen) and Synthemax (Corning), are also available albeit at a high cost.

## THE ROLE OF THE MICROENVIRONMENT AND SYNTHETIC SUBSTRATES

Culture conditions (density of surrounding cells, soluble factors, substrates) should be considered as a whole to capture the complexity of soluble signals and the surrounding environment. Soluble factors such as Wnts and FGFs regulate stem cells self-renewal, some membrane-associated proteins such as cadherins form adherens junctions involved in cell positioning and anchoring (Lutolf et al., 2009) while integrins, bind to other components of the ECM, including fibronectin, vitronectin, laminin, and collagen to promote cell adhesion and differentiation (Fuchs et al., 2004).

Several lines of research have recently attempted to focus on the effect of substrates on the proliferative behavior of stem cells. In tissue stem cells, previous studies have reported that engineered surfaces with precise ligand affinity, density, and tethering, spatial arrangement of surface chemistry, topologies, and matrix stiffness can elicit cell responses ranging from self-renewal to differentiation(McBeath et al., 2004; Engler et al., 2006; Dalby et al., 2007; Khetan and Burdick, 2010; Unadkat et al., 2011; Kilian and Mrksich, 2012; Trappmann et al., 2012; Viswanathan et al., 2012). Such synthetic substrates are valuable tools to dissect cell matrix interactions *in vitro*.

**FIGURE 2:**
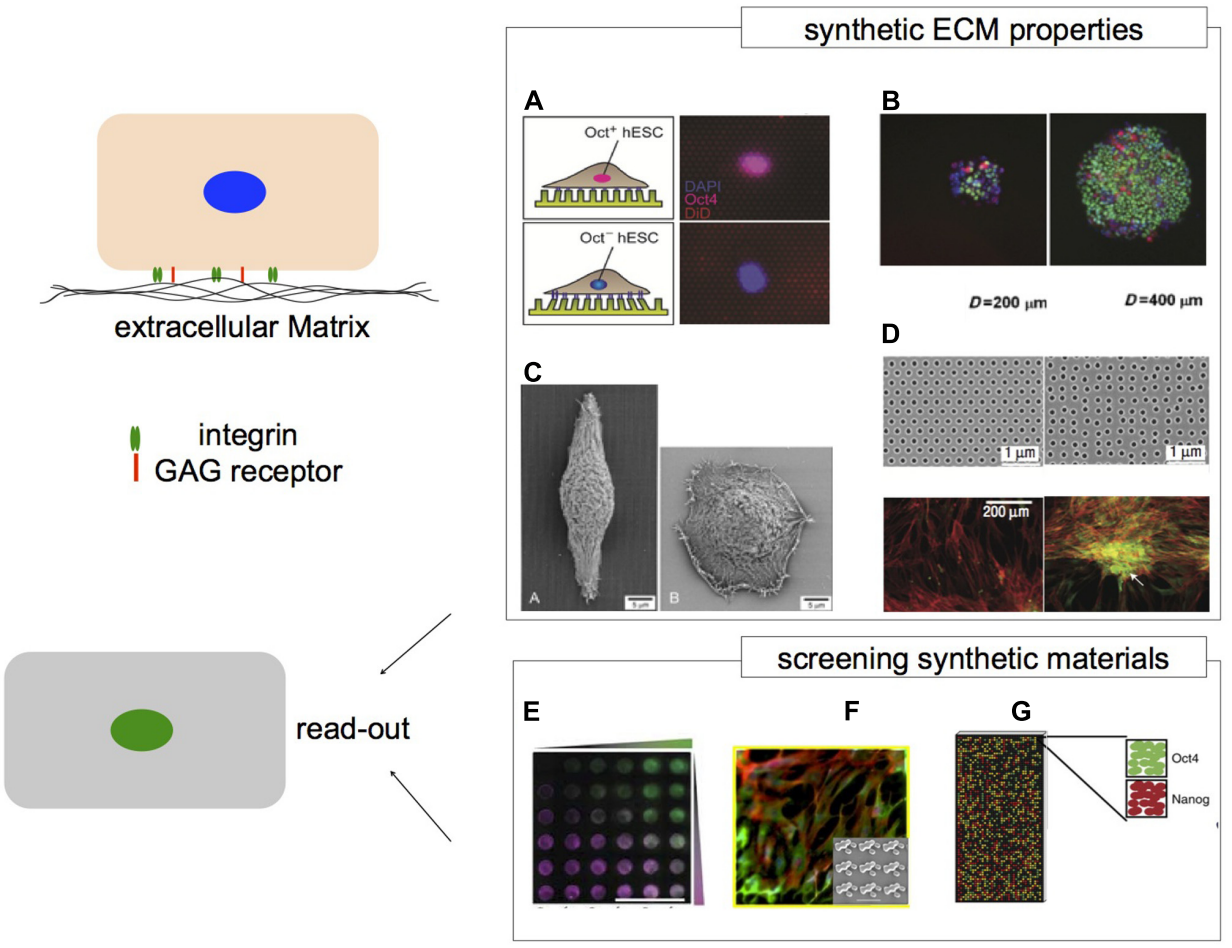
**Microenvironments and their impact on hPS cells.** Cells respond to the surrounding microenvironment via cell–cell contacts and cell–matrix contacts. The ECM provides structural and chemical cues. Synthetic ECM niches can recapitulate aspects of ECM properties to regulate cell behavior. **(A)** Human embryonic stem cells that are Oct4^+^ and Oct4^-^ respond differently to PDMS micro-posts with varied stiffness. **(B)** hES cells grown on larger adhesive islands of 400 μm have greater levels of Oct4 expression via Smad1 signaling. Nuclei depicted in blue, Oct4 in green and pSmad1 in red. **(C)** Micro-grooved substrates (left) can alter epithelial cell morphology by contact guidance compared to flat surfaces (right). **(D)** Symmetry of nanoscale topography with semi-random geometry induces osteogenesis of hMSCs compared to hexagonal geometry. Immunofluorescence represents cytoskeleton in red and osteopontin in green. High content analysis can be applied to screen a wide array of biomaterials to associate specific properties with biological response. Platforms such as **(E)** PEG based hydrogel arrays of protein concentration gradients and PEG stiffness. FITC- and rhodamine labeled BSA gradients are represented in green and magenta, respectively. **(F)** The Topochip platform can be used to vary surface topography of the same material chemistry. **(G)** Spotted polymer arrays of varied substrate mechanical properties for long term culturing of hES cells. **(A)** Image adapted from Sun et al. (2012b). **(B)** Peerani et al. (2007), image adapted with permission from John Wiley and Sons, Copyright 2007. **(C)** Teixeira et al. (2003), image reproduced with permission from *Journal of Cell Science*, Copyright 2003. **(D)** Dalby et al. (2007), image reprinted with permission from Macmillan Publishers Ltd.: *Nature Materials*, Copyright 2007. **(E)** Gobaa et al. (2011), image reprinted with permission from Macmillan Publishers Ltd.: Nature Methods, Copyright 2011. **(F)** Unadkat et al. (2011), image adapted with permission from the *National Academy of Sciences*, Copyright 2011. **(G)** Zhang et al. (2013) image reprinted with permission from Macmillan Publishers Ltd.: *Nature Communications*, Copyright 2013.

These design principles can be extended to determine substrates that contribute to optimal self-renewing conditions as well as materials that direct hPS cells into specific lineage differentiation. In this section we describe the main components of cell–cell and cell–ECM interactions that may guide the design of new synthetic materials. Moreover we discuss matrix properties that can affect hPS cell self-renewal and differentiation and mechanotransduction pathways that are important in these processes. Finally, we review synthetic tools to study cell material interactions, with a view on the potential application for screening materials using hPS and image analysis (**Figure 2**).

### MIMICKING THE EXTRACELLULAR MATRIX (ECM)

Cell–cell and cell–ECM interactions are mediated by integrins, cadherins, or polysaccharides such as glycosaminoglycans (GAGs). These molecules transmit biophysical cues and environmental cues across the cell membrane to intracellular signaling pathways involved in cell fate decisions. Integrins are heterodimeric proteins involved in adhesion and bi-directional signaling containing α and βsubunits. Combinations of these (24 have been described) may take place, determining the ligand specificity and affinity for specific ECM motifs such as the tri-peptide RGD (Arginin–Glycin–Aspartic acid).Their adhesion strength is modulated by activation or clustering, which anchors stem cells to their niche (Ellis and Tanentzapf, 2010). hPS cells have been shown to express a range of integrin chains including α_3_, α_5_, α_6_, and β_1_. Furthermore hES cells also express α_2_, α_11_, α_V_ (Braam et al., 2008; Meng et al., 2010), and hiPS cells, α_7_, α_V_, and β_5_ (Rowland et al., 2010; Jin et al., 2012). GAGs are long unbranched polysaccharides whose chemical functionality determines the type such as heparin, chondroitin or others. GAGs are other mediators of adhesion to the ECM and are abundant on the surface of hPS (Sun and Fu, 2013). Peptide sequences derived from vitronectin (Klim et al., 2010) that bind to heparin have been used as synthetic feeder-free substrates for the maintenance of hPS cell pluripotency and like other ECM components can be readily conjugated to any synthetic surface that are now commercially available (Synthemax, Corning).

### CELL–CELL INTERACTION, CELL SHAPE, CYTOSKELETAL TENSION, AND TOPOGRAPHICAL CUES

Important mediators of cell–cell interactions are proteins from the cadherin family. Cadherins play many roles in cell recognition, cell sorting and strengthening of cell–cell adhesions. They also operate as signaling receptors that modulate cell behavior or drive cell-upon-cell locomotion because they are force-resistant (Niessen et al., 2011). There are three classical types of cadherins that have been most extensively studied: the epithelial (E-cadherins), vascular endothelial (VE-cadherins), and neural (N-cadherins). E-cadherins are involved in calcium-dependent cell–cell adhesion in both epithelial and embryonic stem cells, and are integral for hES cell self-renewal and survival (Xu et al., 2010). E-cadherins are also utilized to identify hES cells as markers of undifferentiated state (Li et al., 2012). They also interact with ROCKs to regulate the function of the actin-cytoskeleton and promote hES cell clonogenicity (Li et al., 2010).

Human mesenchymal stem cells (hMSCs) provided early proof demonstrating that the shape of the substrate used to culture cells could strongly influence cell fate and tissue architecture. A decrease in plating density (or larger fibronectin islands) increased cell spreading and area and induced osteogenic differentiation; conversely an increase in plating density (or smaller fibronectin islands) generated rounded less spread cells and induced adipogenesis. The RhoA-ROCK signaling pathway was implicated in the adipogenic–osteogenic switch. Pharmacological inhibition of RhoA and its effector ROCK has shown to disrupt the cytoskeleton and affect hMSC differentiation mediated by the matrix (McBeath et al., 2004). A more recent study demonstrated that cell shape and cytoskeletal tension rather than the area, dictated hMSC lineage commitment (Kilian et al., 2010). For example, micro-patterned islands with the same area but of different shapes exhibited high or low cytoskeletal contractility resulting in osteogenesis or adipogenesis, respectively.

Though much of what we understand of cell-shape induced differentiation has come from adult stem cells, similarities with hPS cells are beginning to emerge. By patterning Matrigel islands of 200, 400, and 800 μm, bone morphogenic protein (BMP) mediated small body size/mothers against decapentaplegic (Smad1) signaling maintained hES cell pluripotency on the largest islands that supported large, densely packed colonies (Peerani et al., 2007).The dependence of pluripotency on the size of the niche highlights in this case the role of soluble factors secreted by the hPS cells. Mechanotransduction was here not implicated, yet the biophysical signals of the microenvironment in controlling cell shape can be affected by the colony size. UV/ozone patterned vitronectin substrates have been used to study hPS cell shape and morphology at the single cell level both in the presence and absence of ROCKi (Pryzhkova et al., 2013). Patterns that can stimulate cell polarity are crucial to dissect phenomena such as cell shape induced epithelial to mesenchymal transition (EMT), which is a key step in hPS cells differentiation. EMT has also been involved in a study showing that fibroblasts cultured using parallel microgrooves or aligned nanofibers on poly(dimethyl siloxane; PDMS) present an increase in reprogramming efficiency (Downing et al., 2013).

Stem cell growth and differentiation can also be affected by micro- and nano-topographic cues such as grooves, ridges or pits. Grooved and ridged topographies for example, can induce cell alignment and elongation through contact guidance (Teixeira et al., 2003) for a number of specialized cells including differentiation of hES cells (Chan et al., 2013) and hiPS cells toward neuronal lineages (Pan et al., 2013). hES cells cultured on PDMS gratings with 600 nm feature-height and spacing also generated cell alignment in the presence of soluble factors (Gerecht et al., 2007). The polarization of gamma-tubulin complexes (GTCs) on nanotopographies may play a role in mediating topography-induced changes in cell morphology, as GTCs can govern cytoskeletal function and assembly of filamentous actin. However, changes in hES cell shape and morphology governed by actin assembly directing eventual cell fate was not investigated.

Apart from contact guidance, the size, spacing, and orientation of nanotopographies can directly affect cell response resulting from topographies. Surface topography can also alter cell behavior indirectly from changes in the conformation of surface adsorbed proteins. Nanoscale topographies for example, arranged in a square planar geometry induced hES cell differentiation toward the mesenchymal lineage in the absence of growth factors (Kingham et al., 2013). Unlike ECM patterned islands that promote cytoskeletal tension mediated differentiation, it has been suggested that stem cell adhesion to surface topographies is mediated by the modulation of integrin clustering and focal adhesion formation (Biggs et al., 2010; Sun et al., 2012a).

Cell adhesion to the ECM can result in the recruitment, organization, and clustering of integrins and the formation of focal complexes maturing into focal adhesions and providing direct anchorage (via vinculin, talin, and paxillin) to the actin cytoskeleton. Furthermore, integrin mediated adhesion can activate tyrosine kinase and phosphatase signaling to modulate downstream signals that determine cell fate (Vogel and Sheetz, 2006). For example in hES cells cultured on nano-roughened and smooth glass substrates (Chen et al., 2012) maintenance of pluripotency was found to depend on an interplay between focal adhesion formation, cell–cell contacts and cytoskeletal rearrangements mediated by non-muscle myosin IIa (NMMIIa) on flat surfaces. On the other hand, nanotopographic pillars of hexagonal versus honeycomb arrangements (Kong et al., 2013) and nanopillar gradients of varied spacing (Bae et al., 2014) supported Oct4^+^ cells and maintained E-cadherin expression. Moreover, focal adhesion kinase (FAK) inactivation led to a more dynamic reorganization of the cytoskeleton on the topographies of the lowest diameters. Thus, only nascent focal complexes or disrupted focal adhesions rather than mature focal adhesions were observed. These latter studies suggest that selected nanotopographies can be used to maintain pluripotency.

Despite the fact that there is inconsistency in discerning which subset of surface topographical or chemical features eventually dictates hPS cell response, there are early indications that integrins may play a role in hPS cell fate decisions. Moreover, it has been widely demonstrated that integrin-mediated adhesion to the ECM is crucial for hPS cell survival. However, it is still unclear if and how initial adhesion events activate downstream signaling cascades involved in EMT and lineage commitment. FAK activation has been suggested to act upstream of the Rho/ROCK (Bhadriraju et al., 2007) and mitogen-activated protein kinase (MAPK; Salasznyk et al., 2007) signaling pathways, both of which have been implicated in cell shape induced differentiation. Thus, the perturbation of FAK or other focal adhesion complexes or anchor proteins will need to be further explored, in particular its effect on E-cadherin expression and pluripotency.

### hPSC MECHANOSENSING AND SUBSTRATE RIGIDITY

The inherent sensitivity of hPS cells arises from the relatively poor understanding of cell–cell and cell–substrate interactions underlying the maintenance of pluripotency. It is now known that hPS cells undergo dissociation-associated apoptosis and inhibiting RhoA/ROCK mediated, NMMII-dependent cytoskeletal tension enhances hPS cell survival (Ohgushi et al., 2010). As RhoA/ROCK mediated cytoskeletal tension is an important factor in mechanotransduction (McBeath et al., 2004) the cytoskeletal hyperactivation of hPS cells upon dissociation and in conjunction with loss of cell–cell contacts (through loss of E-cadherin expression) suggests that the mechanical properties of the stem cell environment may indeed be key to determining cell fate decisions. P120 catenin, an Armadillo-domain protein implicated in cell–cell adhesion is stabilized by NMMII and this process has been shown to be necessary for E-cadherin dependent mechanical tension and maintenance of pluripotency in hESCs (Li et al., 2010). When cultured on polyacrylamide (PA) gels of 8.5 kPa, continued inhibition of NMMIIa by blebbistatin markedly down-regulated E-Cadherin expression. In another study, hES cells pluripotency was maintained when hES cells were cultured on shorter, stiffer vitronectin coated PDMS microposts and the expression of Oct4 paralleled that of E-cadherin (Sun et al., 2012a). Cell–cell contacts alone, however, may not be involved in sensing matrix rigidity. For example, GAG mediated hES cell adhesion (Klim et al., 2010) to PAgels (Musah et al., 2012) also showed that hES cells preferred adhering to stiffer gels (10 kPa) and when cultured on softer gels (0.7–3 kPa) no longer expressed the pluripotency markers Oct-4 and SSEA-4. Here it was proposed that only the stiff PA gels promoted YAP/TAZ (Yes associated protein/transcriptional coactivator with PDZ binding motif) localization in the nucleus while cells cultured on softer gels exhibited low levels of cytoplasmic (i.e., inactive and the degraded form of) YAP/TAZ. YAP and TAZ serve as mechanosensors and transcriptional regulators required for cell differentiation influenced by substrate stiffness (DuFort et al., 2011) though others have suggested that TAZ functions in hES cell self-renewal (Varelas et al., 2008). The precise mechanisms of YAP/TAZ mechanotransduction in hPS cells is still unknown and more work will be required to unravel a potential role in hPS cell mechanosensing. Adhesion to ECM proteins is highly dependent on integrin-mediated adhesion. It is unclear whether the latter can convey mechanical signals.

### PATHWAYS REGULATING hPSC PROLIFERATIVE BEHAVIOR

Generally, MAPK, protein kinase B (PKB), and nuclear factor κ-light-chain-enhancer of activated B cells (NFκβ) signaling are involved in supporting viability and pluripotency of hPS cells. PKB can cascade through the MAPK signaling pathway resulting in hES cell differentiation. The NFκβ signaling cascade is also involved in cell survival (Armstrong et al., 2006). As mentioned above, inhibition of the ROCK pathway can prevent anoikis of hPS cells when dissociated to single cells; however, manipulation of this pathway has been utilized in other settings. Differentiation can be initiated by RhoA and ROCK activation of myosin light chain kinase (MLCK) controlling the processes of cytoskeletal tension and stress fiber development. This phenomenon has been widely studied in hMSC differentiation. RhoA/ROCK can induce cells to undergo fluid-flow-induced osteogenesis, while on the contrary, the inhibition of this pathway triggers adipogenesis and chondrogenesis (Arnsdorf et al., 2009). The Wnt/β Catenin signaling pathway is needed to preserve and support the pluripotency of hES cells. The actions of Wnts are growth-factor like, and can control asymmetric cell division, cell proliferation, migration, and polarity. Wnts have the ability to enhance the process of somatic cell reprogramming to generate iPS cells. The interaction of β Catenin with transcription factors Sox2, Klf4, and Oct4 can only occur via the Wnt pathway, triggering the upregulation of Nanog, which demonstrates the involvement of this pathway in cell reprogramming, maintenance of the cell in a pluripotent state, and ability for self-renewal (Kuhl and Kuhl, 2013).The Wnt pathway also promotes pluripotency and can be activated by the addition of lithium chloride as in the mTeSR medium formulation. Other signaling pathways involved in pluripotency and self-renewal are transforming growth factor-β (TGF-β), which signals through Smad2/3/4, and FGF2 which signals to its receptor, FGFR to activate the MAPK and PKB pathways (James et al., 2005; Vallier et al., 2005).

### TOOLS TO STUDY SUBSTRATE EFFECTS ON CELL BEHAVIOR

Advances in synthesis and fabrication techniques have allowed for a wide range of materials suitable for applications in cell biology. Fabrication of synthetic matrices may be produced from either “top-down” or “bottom-up” approaches. As this encompasses a large body of work, the reader is directed to several reviews that summarize the types of materials used in matrix mediated stem cell differentiation (Stevens and George, 2005; Sands and Mooney, 2007; Lutolf, 2009). The most common of these materials and fabrication techniques used in cell biology for exploring the cell–materials interface are briefly discussed here.

Hydrogels are polymer networks mimicking many aspects of the native ECM and are readily hydrated. They can be easily manufactured and can be tuned to the desired elastic and viscous moduli, making them attractive for studying mechanotransduction. Most hydrogels are often composed of cell/protein inert chemistries, for example poly(ethylene glycol; PEG) and require functionalization to promote cell–material interactions. The surface functionality of PEG or PEG macromer hydrogels, can be modified by conjugating peptides or proteins to the backbone of PEG for example, via Michael-type additions requiring PEG macromers end functionalized for example with acrylate or vinyl sulfone groups that readily react with thiols (Metters and Hubbell, 2005). Other methods of conjugation may also be implemented (Liu et al., 2010). Such hydrogels can also be modulated in stiffness by tuning the cross-linking density or the molecular weight of the PEG macromer. In general, hydrogels offer an easy starting point for developing defined synthetic niches. For example, the covalent attachment of peptides such as the integrin binding sequence RGD or GAGs (Klim et al., 2010; Musah et al., 2012) or matrix metalloproteinases (MMPs; Jang et al., 2013) to result in 2D or 3D scaffolds have been used for hPS cell propagation. Other hydrogels based on hyaluronic acid (Gerecht et al., 2007) that can bind to cells via CD44 surface receptors have also been used to culture hES cells albeit using feeder conditioned medium.

Topographical features have been fabricated using a range of lithographic techniques such as electron beam-, photo/UV- or dip-pen lithography or through microcontact printing. The advantages of these methods are that features can be fabricated in a highly reproducible manner and can be highly ordered spatially. Microcontact printing (μ-CP) utilizes an elastomeric PDMS stamp consisting of the desired features, which is then used to transfer “inked” material onto a substrate. In this way, many materials such as individual ECM proteins are patterned for single cell studies (Mrksich and Whitesides, 1995; Ruiz and Chen, 2007). Photo/UV- and electron beam-lithographies on the other hand can be used to produce topographical features such as grooves, pits, and islands in the micro- and nano-meter length scales that can be ordered or disordered over large areas providing a plethora of tools for studying fundamental cell biology.

### FINDING THE RIGHT NICHE: SCREENING BIOMATERIALS USING hPS CELLS

As hinted above, it is now increasingly accepted that biophysical cues arise not only from surface chemistry but also from topography. In combination with soluble factors, these can have a profound influence on determining cell fate of hPS cells. Engineered biomaterials are therefore studied to recapitulate biological complexity (i.e., combining key components of matrix properties, heterogeneity, and complexity) and understand the relationships between the physical and chemical properties of the material and its interaction with cells. To this end, screening approaches with materials in high throughput or “materi-omics” (Cranford et al., 2013) have been attempted such as the “Topochip” platform (Unadkat et al., 2011). In this study, photolithography techniques were used to generate more than 2000 unique micro-scale topographies (though the possibilities are far greater) by combining the primitive shapes; circles, rectangles, and triangles. Here, surfaces that promoted osteogenic differentiation of cultured hMSCs in the absence of soluble growth factors were investigated. Protein-based microarrays by robotically spotting various ECM proteins have been previously used to study hES cell–matrix interactions (Flaim et al., 2005). Additionally, others (Gobaa et al., 2011) have developed a hydrogel microwell array that combines both and physical properties to encapsulate both adherent and non-adherent cells. Such platforms have been used to probe cell–cell interactions and cell–materials interactions that drive osteo- and adipogenic differentiation of hMSCs and may be adapted to the study of microenvironments affecting hPS. The first progress in this direction involves the use of polymer arrays with inkjet printing to combine acrylate and acrylamide monomers and generate thermo-responsive hydrogels (Zhang et al., 2013). Such stimuli responsive matrices were used to mechanically disperse cells as an alternative to enzymatic dissociation while supporting hES cell proliferation and pluripotency in culture.

The increased throughput and the development of comprehensive structure–function methodologies in these cutting edge studies will allow quicker identification of the most relevant synthetic substrates for specific responses (Mei et al., 2010; Saha et al., 2011). Although many materials technologies have advanced to produce a vast selection of topographies, chemistries, and combinations, a challenge currently faced is how to predict and quantify stem cell responses at the single cell levelusing engineered microenvironments (Treiser et al., 2010; Vega et al., 2012).

## STRATEGIES FOR HIGH CONTENT ANALYSIS AND DISEASE MODELING

**FIGURE 3:**
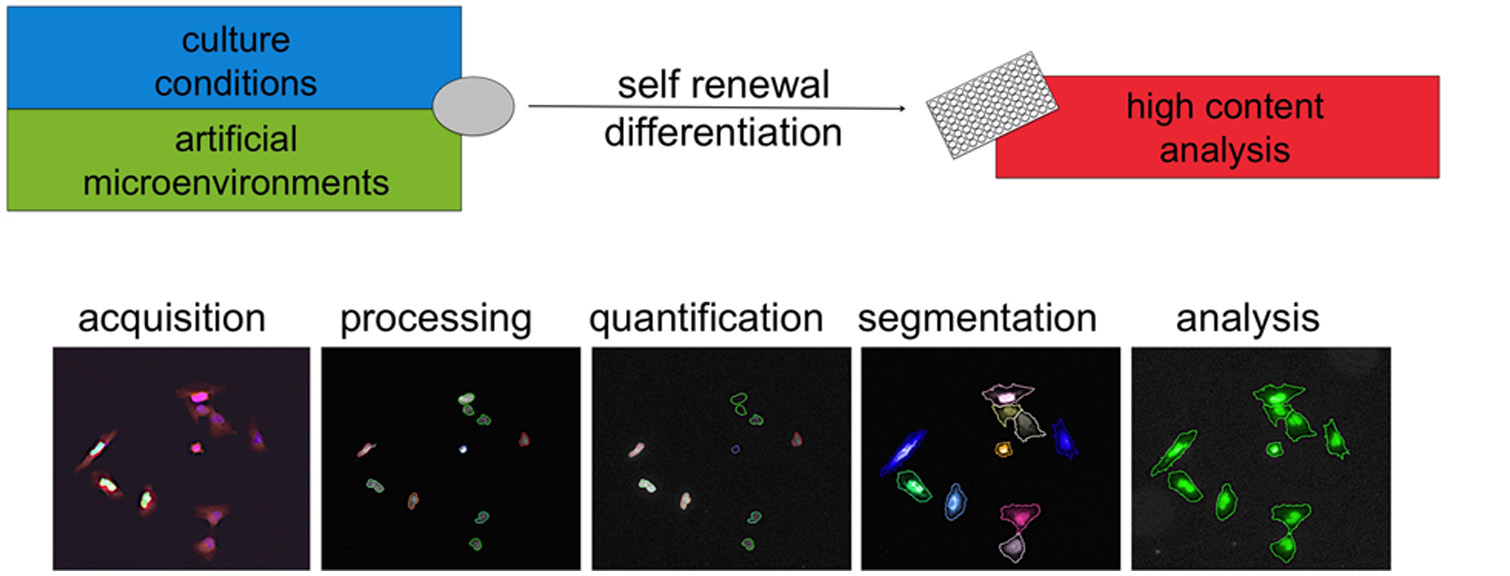
**High content analysis.** High content analysis involves a series of steps to convert images into data. Cells in different conditions are imaged via microscopy. Images are then processed (for example in contrast) and then analyzed using computer algorithms. Segmentation refers to the definition based on pixels intensity of “objects” such as cells, nuclei, or other subcellular structures. Via cytochemistry, the expression of specific markers can be elicited and the levels quantified. These measures can be combined with morphological parameters and objects can be included or excluded from further analysis. This approach is now increasingly applied to the use of new artificial microenvironments to develop specific conditions allowing self-renewal or differentiation. This is likely to synergize with the advances in the understanding of human pluripotent stem cells culture and bring fast and substantial contribution to disease modeling and drug discovery.

Together with cell culture and liquid handling, the technologies available for microscopy, image analysis, and computing have undergone an extremely rapid progress in recent times. Collectively, the confluence of outputs from such distinct fields has brought to life the discipline of high content analysis (HCA). Cells can be readily examined in real time or in cytochemistry endpoint assays. Acquired images are processed and groups of pixels are computationally segmented into defined “objects”capturing imaged cells, nuclei, or subcellular organelles (**Figure 3**).This allows quantification of proliferative behavior, morphology changes, and expression of proteins such as lineage or functional markers. Importantly this can now happen upon exposure to up to several thousands conditions per week so that the term high throughput can be appropriately used for HCA approaches as well. Complex datasets are acquired and interrogated using proprietary or open source computational tools as detailed elsewhere (Singh et al., 2014). The value of these methods in discovering new chemical entities has been demonstrated (Swinney and Anthony, 2011).

Biologically significant assays may help ensure that toxic or non-effective drugs fail *in vitro*, in the pre-clinical phases and not in the clinic, with huge benefits for the cost of the discovery process and for the patients. The field has developed substantially using cancer cell lines, typically well suited for cell based assays but of unclear biological relevance (Wilding and Bodmer, 2014). On the other hand, more relevant primary cells are often not suitable due to phenomena such as replicative senescence, differentiation, spontaneous immortalization or transformation in culture. The focus on identification of optimal cell types for HCA has highlighted hPS cells and their derivatives as an attractive alternative for a number of reasons. First, the capability to self-renew and generate a consistent starting population of cells over a number of passages reduces variability of the starting population. Secondly, together with robust differentiation protocols, hPS cells can be used to produce the high number of progenitors or differentiated cells required for screening. Additionally, patient-derived iPS cells offer unique tools to study the range of physio-pathological mechanisms involved in selected diseases at the cellular level and to identify drugs that benefit specific cohorts of patients. It is also worth to note that in some cases, diseases can present with a block of differentiation (Kuhlmann et al., 2008). Assays that are built around the specific differentiation protocols may in principle be used to screen for drugs that bypass the differentiation blocks to develop therapeutics.

### HIGH CONTENT ANALYSIS APPROACHES USING HUMAN PLURIPOTENT STEM CELLS

We will not discuss further studies aimed at isolating compounds that improve reprogramming as these are discussed elsewhere (Chen et al., 2010b; Yang et al., 2011; Li and Rana, 2012). In reviewing HCA screens using hPS cells with a reasonable throughput we will start by focusing on survival and self-renewal (**Table 1**). Distinguishing between effects that are limited to prolonged survival or true long-term maintenance of self-renewal can be difficult. Visualizing hPS cell colonies formed on feeders, TRA-1-60 staining and DNA dyes were chosen as a suitable marker of pluripotency (Barbaric et al., 2010a,b). Imaging pipelines were here used to “erode” and “dilate” in parallel the segmented nuclei in order to accurately quantify on one hand the percentage of undifferentiated cells and to remove on the other from the analysis confusing data regarding feeder cells (Barbaric et al., 2011). This strategy isolated the antihypertensive drug pinacidil as a promoter of hES cells survival. Independently, the same molecule was found using hES cells feeder free with conditioned medium; here the authors showed pinacidil as well as other compounds to be structurally related to classic ROCK inhibitors (Andrews et al., 2010). Similar approaches have been tried elsewhere and identified compounds that improved survival by inhibiting ROCK or protein kinase C (Damoiseaux et al., 2009; Sherman et al., 2011). Notably, automation and culture in 384 wells was applied to hES cell culture in a single-cell dissociation protocol using conditioned medium and quantitation of the pluripotency marker Oct4 together with a nuclear dye to isolate compounds promoting differentiation or expansion (Desbordes et al., 2008; Desbordes and Studer, 2013).In line with the importance of cell–cell contacts and cell–ECM contacts described above, it is interesting to note that a central role for signaling involving E-cadherin, RhoA/ROCK pathway and integrins in survival has also been proposed based on HCA screening (Xu et al., 2010). A different angle was taken in (Ben-David et al., 2013) to use HCA to identify compounds that selectively eliminate hPS over differentiated cells for future cell therapeutic application to mitigate the risk of teratomas. Over 50,000 compounds were screened against undifferentiated hES on matrigel with mTeSR1. Cytotoxic compounds were then tested on nine different cell types with very stringent criteria. A selective inhibitor, PluriSIn #1 was identified this way. Very few attempts have been reported to challenge hPS against a substantial number of materials as screening conditions. Among these, in Mei et al. (2010), combinations of monomers were screened on a primary array for colony formation using transgenic Oct4-GFP hES cells flow sorted and seeded in near-clonal density. The results were then compared with an analysis of physical characteristics of the materials. Surface roughness, indentation, elastic modulus, and wettability were included. Self-renewal ability of cells was showed to be dependent on adsorbed proteins, surface chemistry and the geometry of the spot the cells were occupying. Several hit polymers coated with vitronectin in mTeSR1 were suggested as the most advanced culture conditions. It is likely that future studies will include chemistries and materials as it has been attempted for MSC. Altogether, the majority of these studies use end-point assays that require fixation and staining of cells. An alternative or complementary HCA tool is live imaging. This has proved effective in iPS-derived neural cells (Danovi et al., 2010) and also as a tool to distinguish cells that are genuinely reprogrammed (Chan et al., 2009). Because of the substantial improvement in the field and its power, we predict that live imaging will raise in importance using hPS cells for screening and will be applied in synergy with end-point assays and the use of artificial microenvironments to model diseases.

**Table 1:** High content analysis chemical screens using human pluripotent stem cells.

Source of cells	Culture conditions	Number of cells	Read out	Imaging device	Number of conditions	Proposed pathways	Reference
hES (SA461)	Single cells, fibronectin, FF, MEF-CM	5000 per 96 well	“Percent Activation” from DAPI	InCell 1000	1200+, 4100+, 15000	ROCK	Andrews et al. (2010)
hES (Shef4)	Colonies on MEFs	6000 per 96 well	Tra 1-60, Hoechst	InCell 1000	1040	ROCK	Barbaric et al. (2010b)
hES (HSF1, H9)	Single cells on MEFs, gelatin, DM	Approx 5000 per 384 well	Oct4, Hoechst	Image Xpress micro	1280+, 504	ROCK and PKB	Damoiseaux et al. (2009)
hES (H9)	Single cells on Matrigel, MEF-CM, automation	6000 per 384 well	Oct4, Hoechst	Incell 3000	2880	TGFβ, wnt, FGF2	Desbordes et al. (2008)
hES (BG01, WIBR3)	Single cells on polymer, MEF-CM	Low density, 40 cells mm^-2^	Oct 4, SSEA-4	iCys laser scanning, AFM	496 (materials)	–	Mei et al. (2010)
hES (HEUS9)	Single cells on Matrigel, DM	4000 per 384 well	ALP, compact colony morphology	Inverted micro-scope	50,000	E-cadherin	Xu et al. (2010)

*Abbreviations: PKC, protein kinase C; AP, alkaline phosphatase; MEF, mouse embryonic fibroblast; CM, conditioned medium; FF, feeder free; DM, defined medium; FGF-2, fibroblast growth factor 2.*

### HCA, hPS CELLS, DISEASE MODELING, AND DRUG DISCOVERY

Disease modeling is possibly the most immediate potential application of hPS cells to therapy. The majority of studies have used selected small number of compounds in hypothesis testing experiments. We will cover selected cases and refer the reader to recent reviews for a more exhaustive perspectives (Maury et al., 2012; Robinton and Daley, 2012) and for neural diseases (Xu and Zhong, 2013; Imaizumi and Okano, 2014).

Seminal studies on Spinal Muscular Atrophy (Ebert et al., 2009) offered the proof of principle that it is possible to obtain reprogrammed cells from patients suffering a specific disease. The authors also reported a disease phenotype: patients-derived cells proved impaired in their neuronal differentiation and gave rise to neurons that were smaller in size, lacking the SMN protein and specific nuclear gems structures. Selected compounds, valproic acid and tobramycin could rescue this phenotype. Ectopic expression of SMN was later shown to rescue a similar phenotype (Chang et al., 2011). Amyotrophic Lateral Sclerosis was also the subject of recent attention. Screens using motoneurons from both wild-type and mutant SOD1 mouse model allowed the isolation of hits compounds. Kenpaullone was then validated using cells differentiated from patients-derived iPS cells and improved the survival of motoneurons more than compounds that recently failed in ALS clinical trials (Yang et al., 2013; see also Makhortova et al., 2011; Burkhardt et al., 2013; Chestkov et al., 2014).

Alzheimer has also been modeled (Yagi et al., 2011) and drug screening platforms have been proposed (Yahata et al., 2011; Xu et al., 2013). A recent study pointed toward the limitation of classic HCA assays in 2D for this disease. Neurons from iPS-derived neuroepithelial cells were derived. From these, the detection of canonical features of the disease was possible in 3D but not in classic assays (Zhang et al., 2014). Schizophrenia has been investigated extensively in terms of its genetic etiology with no conclusive consensus. A number of differences in phenotype and gene expression were observed in cells derived from patients versus healthy individuals (Brennand et al., 2011). Importantly, key cellular and molecular traits were shown to ameliorate with an antipsychotic, loxapine. Other important studies were reported for dementia (Almeida et al., 2012) and familial dysautonomia (Lee et al., 2009, 2012). Parkinson’s disease (PD) is particularly difficult to model using current hiPS cells. Cells reprogrammed from a patient that showed triplication of the alpha-synuclein locus and their comparison with those derived from an unaffected first-degree relative represent a significant step forward (Devine et al., 2011). The pathological aggregation of alpha-synuclein present in PD-affected neurons was recapitulated in iPS-derived cells. Then the authors turned to yeast to identify early pathogenic phenotypes and showed that a small molecule (NAB2) and its target Nedd4 could rescue the disease phenotype (Chung et al., 2013). Another important study focusing on PD offers a fascinating insight in the differences and similarities between senescence and aging while proposing an interesting option to model late-onset diseases overcoming the “rejuvenation” that is triggered with reprogramming (Miller et al., 2013).

Beside neurological diseases, several attempts modeling cardiac diseases were undertaken. Cardiomyocytes derived from patients carrying LEOPARD syndrome, an autosomal dominant developmental disorder (Carvajal-Vergara et al., 2010) showed larger higher sarcomeric organization and preferential nuclear localization of NFATC4 in the nucleus when compared with cardiomyocytes derived from hES cells or hiPS cells from healthy sibling donors. Congenital long QT syndrome is a familiar arrhythmia. Cardiomyocytes derived from hiPS from type 2 long QT syndrome patients showed significant prolongation of the action-potential duration and were used to evaluate the effects of channel blocker drugs (Itzhaki et al., 2011) reviewed in (Friedrichs et al., 2013). In some cases gene abnormalities have been corrected in hiPS cells opening interesting prospects for both cell therapy and disease modeling (Yusa et al., 2011; see also Choi et al., 2013). Despite the impressive progress in the field, very few studies have successfully recreated features of the disease in a cell-based assay robust enough to be screened with a substantial number of conditions on hiPS-derived cells.

### CONNECTING THE DOTS

In order to allow HCA, several requisites are necessary as summarized in (Engle and Vincent, 2014). Cell robustness and reproducibility, in vitro differentiation, reasonable throughput, relevance, assay characteristics, screening cascade design are all of paramount importance to achieve meaningful disease modeling. We envision that in the near future, synthetic materials and sophisticated HCA analysis including bright field label free live imaging will enrich the palette of tools available. There is a growing awareness that the understanding of pluripotent stem cells, the definition of culture conditions, the engineering of optimal substrates and the development of appropriate HCA pipelines can be combined toward disease modeling.

Recently, several projects have been launched aimed at establishing multidisciplinary frameworks to characterize several hundreds lines derived from patients and/or healthy individuals. Some examples include the California Institute for Regenerative Medicine (CIRM), the New York Stem Cell Foundation (NYSCF), the Harvard Stem Cell Institute iPS Core Facility, the hPS cell database at the National Institute of Health (StemCellDB NIH), all based in the United States. In Asia, the China IPSCs program and the Japan Science and Technology (JST) agency among others also hold stem cells programs. European initiatives include the European Bank for induced pluripotent stem cells (EBiSC), The Innovative Medicine Initative (IMI)-funded StemBancc, and the hiPS cells initiative (HipSci). Combined these programs will generate hiPS cell lines from approximately 10,000 individuals. HipSci was established in November 2012, headed by Prof. Fiona Watt (London) and Prof. Richard Durbin (Cambridge). Engagement of the clinical genetics community, open access model of data sharing and collaborative cell phenotyping are key features of the project. A bank of several hundred iPS cell lines is being generated and extensive genome, epigenome, proteome, and phenotype analysis is being carried out at the partnering centers. The project aims to develop a baseline analysis for iPS lines from healthy individuals and valuable assays for rare diseases for which calls for proposals have been launched.

In conclusion, the knowledge required to capture the complexity of the field is broad in spectrum; the technology needs to remain focused on the development of relevant complementary tools while exploring the synergies between these. Our goal in this review is to transmit a sense of the diverse backgrounds required for this purpose. An impressive set of resources is being devoted through innovative platforms and bridging between governmental, academic, and commercial partners to expand the core of competencies around stem cells, artificial microenvironments, and HCA. Our hope is this will soon allow to harness the full potential of hPS cells to model diseases and to develop therapeutics.

## Conflict of Interest Statement

The authors declare that the research was conducted in the absence of any commercial or financial relationships that could be construed as a potential conflict of interest.

## References

[B1] Almeida S. Zhang Z. Coppola G. Mao W. Futai K. Karydas A. (2012). Induced pluripotent stem cell models of progranulin-deficient frontotemporal dementia uncover specific reversible neuronal defects. *Cell Rep.* 2 789–798 10.1016/j.celrep.2012.09.007 23063362 PMC3532907

[B2] Andrews P. D. Becroft M. Aspegren A. Gilmour J. James M. J. McRae S. (2010). High-content screening of feeder-free human embryonic stem cells to identify pro-survival small molecules. *Biochem. J.* 432 21–33 10.1042/BJ20101022 20854259

[B3] Armstrong L. Hughes O. Yung S. Hyslop L. Stewart R. Wappler I. (2006). The role of PI3K/AKT, MAPK/ERK and NFkappabeta signalling in the maintenance of human embryonic stem cell pluripotency and viability highlighted by transcriptional profiling and functional analysis. *Hum. Mol. Genet.* 15 1894–1913 10.1093/hmg/ddl112 16644866

[B4] Arnsdorf E. J. Tummala P. Kwon R. Y. Jacobs C. R. (2009). Mechanically induced osteogenic differentiation– the role of RhoA, ROCKII and cytoskeletal dynamics. *J. Cell Sci.* 122 546–553 10.1242/jcs.036293 19174467 PMC2714434

[B5] Bae D. Moon S. H. Park B. G. Park S. J. Jung T. Kim J. S. (2014). Nanotopographical control for maintaining undifferentiated human embryonic stem cell colonies in feeder free conditions. *Biomaterials* 35 916–928 10.1016/j.biomaterials.2013.10.031 24183167

[B6] Barbaric I. Gokhale P. J. Andrews P. W. (2010a). High-content screening of small compounds on human embryonic stem cells. *Biochem. Soc. Trans.* 38 1046–1050 10.1042/BST0381046 20659001

[B7] Barbaric I. Gokhale P. J. Jones M. Glen A. Baker D. Andrews P. W. (2010b). Novel regulators of stem cell fates identified by a multivariate phenotype screen of small compounds on human embryonic stem cell colonies. *Stem Cell Res.* 5 104–119 10.1016/j.scr.2010.04.006 20542750

[B8] Barbaric I. Jones M. Harley D. J. Gokhale P. J. Andrews P. W. (2011). High-content screening for chemical modulators of embryonal carcinoma cell differentiation and survival. *J. Biomol. Screen.* 16 603–617 10.1177/1087057111406547 21593487

[B9] Bedzhov I. Zernicka-Goetz M. (2014). Self-organizing properties of mouse pluripotent cells initiate morphogenesis upon implantation. *Cell* 156 1032–1044 10.1016/j.cell.2014.01.023 24529478 PMC3991392

[B10] Beers J. Gulbranson D. R. George N. Siniscalchi L. I. Jones J. Thomson J. A. (2012). Passaging and colony expansion of human pluripotent stem cells by enzyme-free dissociation in chemically defined culture conditions. *Nat. Protoc.* 7 2029–2040 10.1038/nprot.2012.130 23099485 PMC3571618

[B11] Ben-David U. Gan Q. F. Golan-Lev T. Arora P. Yanuka O. Oren Y. S. (2013). Selective elimination of human pluripotent stem cells by an oleate synthesis inhibitor discovered in a high-throughput screen. *Cell Stem Cell* 12 167–179 10.1016/j.stem.2012.11.015 23318055

[B12] Bhadriraju K. Yang M. Alom Ruiz S. Pirone D. Tan J. Chen C. S. (2007). Activation of ROCK by RhoA is regulated by cell adhesion, shape, and cytoskeletal tension. *Exp. Cell Res.* 313 3616–3623 10.1016/j.yexcr.2007.07.002 17673200 PMC2064860

[B13] Biggs M. J. Richards R. G. Dalby M. J. (2010). Nanotopographical modification: a regulator of cellular function through focal adhesions. *Nanomedicine* 6 619–633 10.1016/j.nano.2010.01.009 20138244 PMC2965469

[B14] Braam S. R. Zeinstra L. Litjens S. Ward-van Oostwaard D. van den Brink S. van Laake L. (2008). Recombinant vitronectin is a functionally defined substrate that supports human embryonic stem cell self-renewal via alphavbeta5 integrin. *Stem Cells* 26 2257–2265 10.1634/stemcells.2008-0291 18599809

[B15] Brennand K. J. Simone A. Jou J. Gelboin-Burkhart C. Tran N. Sangar S. (2011). Modelling schizophrenia using human induced pluripotent stem cells. *Nature* 473 221–225 10.1038/nature09915 21490598 PMC3392969

[B16] Burkhardt M. F. Martinez F. J. Wright S. Ramos C. Volfson D. Mason M. (2013). A cellular model for sporadic ALS using patient-derived induced pluripotent stem cells. *Mol. Cell. Neurosci.* 56 355–364 10.1016/j.mcn.2013.07.007 23891805 PMC4772428

[B17] Carvajal-Vergara X. Sevilla A. D’Souza S. L. Ang Y. S. Schaniel C. Lee D. F. (2010). Patient-specific induced pluripotent stem-cell-derived models of LEOPARD syndrome. *Nature* 465 808–812 10.1038/nature09005 20535210 PMC2885001

[B18] Chan E. M. Ratanasirintrawoot S. Park I. H. Manos P. D. Loh Y. H. Huo H. (2009). Live cell imaging distinguishes bona fide human iPS cells from partially reprogrammed cells. *Nat. Biotechnol.* 27 1033–1037 10.1038/nbt.1580 19826408

[B19] Chang T. Zheng W. Tsark W. Bates S. Huang H. Lin R. J. (2011). Brief report: phenotypic rescue of induced pluripotent stem cell-derived motoneurons of a spinal muscular atrophy patient. *Stem Cells* 29 2090–2093 10.1002/stem.749 21956898

[B20] Chan L. Y. Birch W. R. Yim E. K. Choo A. B. (2013). Temporal application of topography to increase the rate of neural differentiation from human pluripotent stem cells. *Biomaterials* 34 382–392 10.1016/j.biomaterials.2012.09.033 23083932

[B21] Chen A. K. Chen X. Choo A. B. Reuveny S. Oh S. K. (2010a). Expansion of human embryonic stem cells on cellulose microcarriers. *Curr. Protoc. Stem Cell Biol.* Chap.1 Unit 1C, 11. 10.1002/9780470151808.sc01c11s14 20814936

[B22] Chen T. Yuan D. Wei B. Jiang J. Kang J. Ling K. (2010b). E-cadherin-mediated cell–cell contact is critical for induced pluripotent stem cell generation. *Stem Cells* 28 1315–1325 10.1002/stem.456 20521328

[B23] Chen K. G. Mallon B. S. McKay R. D. Robey P. G. (2014). Human pluripotent stem cell culture: considerations for maintenance, expansion, and therapeutics. *Cell Stem Cell* 14 13–26 10.1016/j.stem.2013.12.005 24388173 PMC3915741

[B24] Chen W. Villa-Diaz L. G. Sun Y. Weng S. Kim J. K. Lam R. H. (2012). Nanotopography influences adhesion, spreading, and self-renewal of human embryonic stem cells. *ACS Nano* 6 4094–4103 10.1021/nn3004923 22486594 PMC3358529

[B25] Chestkov I. V. Vasilieva E. A. Illarioshkin S. N. Lagarkova M. A. Kiselev S. L. (2014). Patient-specific induced pluripotent stem cells for SOD1-associated amyotrophic lateral sclerosis pathogenesis studies. *Acta Nat.* 6 54–60 PMC3999466 24772327

[B26] Choi S. M. Kim Y. Shim J. S. Park J. T. Wang R. H. Leach S. D. (2013). Efficient drug screening and gene correction for treating liver disease using patient-specific stem cells. *Hepatology* 57 2458–2468 10.1002/hep.26237 23325555 PMC3633649

[B27] Chung C. Y. Khurana V. Auluck P. K. Tardiff D. F. Mazzulli J. R. Soldner F. (2013). Identification and rescue of alpha-synuclein toxicity in Parkinson patient-derived neurons. *Science* 342 983–987 10.1126/science.1245296 24158904 PMC4022187

[B28] Cranford S. W. de Boer J. van Blitterswijk C. Buehler M. J. (2013). Materiomics: an -omics approach to biomaterials research. *Adv. Mater.* 25 802–824 10.1002/adma.201202553 23297023

[B29] Dalby M. J. Gadegaard N. Tare R. Andar A. Riehle M. O. Herzyk P. (2007). The control of human mesenchymal cell differentiation using nanoscale symmetry and disorder. *Nat. Mater.* 6 997–1003 10.1038/nmat2013 17891143

[B30] Damoiseaux R. Sherman S. P. Alva J. A. Peterson C. Pyle A. D. (2009). Integrated chemical genomics reveals modifiers of survival in human embryonic stem cells. *Stem Cells* 27 533–542 10.1634/stemcells.2008-0596 19074420 PMC3962308

[B31] Danovi D. Falk A. Humphreys P. Vickers R. Tinsley J. Smith A. G. (2010). Imaging-based chemical screens using normal and glioma-derived neural stem cells. *Biochem. Soc. Trans.* 38 1067–1071 10.1042/BST0381067 20659005

[B32] Desbordes S. C. Placantonakis D. G. Ciro A. Socci N. D. Lee G. Djaballah H. (2008). High-throughput screening assay for the identification of compounds regulating self-renewal and differentiation in human embryonic stem cells. *Cell Stem Cell* 2 602–612 10.1016/j.stem.2008.05.010 18522853 PMC2756729

[B33] Desbordes S. C. Studer L. (2013). Adapting human pluripotent stem cells to high-throughput and high-content screening. *Nat. Protoc.* 8 111–130 10.1038/nprot.2012.139 23257981

[B34] Devine M. J. Ryten M. Vodicka P. Thomson A. J. Burdon T. Houlden H. (2011). Parkinson’s disease induced pluripotent stem cells with triplication of the alpha-synuclein locus. *Nat. Commun.* 2:440 10.1038/ncomms1453 PMC3265381 21863007

[B35] Downing T. L. Soto J. Morez C. Houssin T. Fritz A. Yuan F. (2013). Biophysical regulation of epigenetic state and cell reprogramming. *Nat. Mater.* 12 1154–1162 10.1038/nmat3777 24141451 PMC9675045

[B36] DuFort C. C. Paszek M. J. Weaver V. M. (2011). Balancing forces: architectural control of mechanotransduction. *Nat. Rev. Mol. Cell Biol.* 12 308–319 10.1038/nrm3112 21508987 PMC3564968

[B37] Ebert A. D. Yu J. Rose F. F. Jr. Mattis V. B. Lorson C. L. Thomson J. A. (2009). Induced pluripotent stem cells from a spinal muscular atrophy patient. *Nature* 457 277–280 10.1038/nature07677 19098894 PMC2659408

[B38] Ellis S. J. Tanentzapf G. (2010). Integrin-mediated adhesion and stem-cell–niche interactions. *Cell Tissue Res.* 339 121–130 10.1007/s00441-009-0828-4 19588168

[B39] Engle S. J. Vincent F. (2014). Small molecule screening in human induced pluripotent stem cell-derived terminal cell types. *J. Biol. Chem.* 289 4562–4570 10.1074/jbc.R113.529156 24362033 PMC3931017

[B40] Engler A. J. Sen S. Sweeney H. L. Discher D. E. (2006). Matrix elasticity directs stem cell lineage specification. *Cell* 126 677–689 10.1016/j.cell.2006.06.044 16923388

[B41] Fischer B. Bavister B. D. (1993). Oxygen tension in the oviduct and uterus of rhesus monkeys, hamsters and rabbits. *J. Reprod. Fertil.* 99 673–679 10.1530/jrf.0.0990673 8107053

[B42] Flaim C. J. Chien S. Bhatia S. N. (2005). An extracellular matrix microarray for probing cellular differentiation. *Nat. Methods* 2 119–125 10.1038/nmeth736 15782209

[B43] Forristal C. E. Wright K. L. Hanley N. A. Oreffo R. O. Houghton F. D. (2010). Hypoxia inducible factors regulate pluripotency and proliferation in human embryonic stem cells cultured at reduced oxygen tensions. *Reproduction* 139 85–97 10.1530/REP-09-0300 19755485 PMC2791494

[B44] Forsyth N. R. Musio A. Vezzoni P. Simpson A. H. Noble B. S. McWhir J. (2006). Physiologic oxygen enhances human embryonic stem cell clonal recovery and reduces chromosomal abnormalities. *Cloning Stem Cells* 8 16–23 10.1089/clo.2006.8.16 16571074

[B45] Friedrichs S. Malan D. Sasse P. (2013). Modeling long QT syndromes using induced pluripotent stem cells: current progress and future challenges. *Trends Cardiovasc. Med.* 23 91–98 10.1016/j.tcm.2012.09.006 23266156

[B46] Fuchs E. Tumbar T. Guasch G. (2004). Socializing with the neighbors: stem cells and their niche. *Cell* 116 769–778 10.1016/S0092-8674(04)00255-7 15035980

[B47] Gafni O. Weinberger L. Mansour A. A. Manor Y. S. Chomsky E. Ben-Yosef D. (2013). Derivation of novel human ground state naive pluripotent stem cells. *Nature* 504 282–286 10.1038/nature12745 24172903

[B48] Gerecht S. Bettinger C. J. Zhang Z. Borenstein J. T. Vunjak-Novakovic G. Langer R. (2007). The effect of actin disrupting agents on contact guidance of human embryonic stem cells. *Biomaterials* 28 4068–4077 10.1016/j.biomaterials.2007.05.027 17576011 PMC2257875

[B49] Gobaa S. Hoehnel S. Roccio M. Negro A. Kobel S. Lutolf M. P. (2011). Artificial niche microarrays for probing single stem cell fate in high throughput. *Nat. Methods* 8 949–955 10.1038/nmeth.1732 21983923

[B50] Greber B. Wu G. Bernemann C. Joo J. Y. Han D. W. Ko K. (2010). Conserved and divergent roles of FGF signaling in mouse epiblast stem cells and human embryonic stem cells. *Cell Stem Cell* 6 215–226 10.1016/j.stem.2010.01.003 20207225

[B51] Haupt S. Grutzner J. Thier M. C. Kallweit T. Rath B. H. Laufenberg I. (2012). Automated selection and harvesting of pluripotent stem cell colonies. *Biotechnol. Appl. Biochem.* 59 77–87 10.1002/bab.1014 23586788

[B52] Heng B. C. Liu H. Cao T. (2004). Feeder cell density–a key parameter in human embryonic stem cell culture. *In Vitro Cell Dev. Biol. Anim.* 40 255–257 10.1290/0407052.1 15723559

[B53] Hussain W. Moens N. Veraitch F. S. Hernandez D. Mason C. Lye G. J. (2013). Reproducible culture and differentiation of mouse embryonic stem cells using an automated microwell platform. *Biochem. Eng. J.* 77 246–257 10.1016/j.bej.2013.05.008 23956681 PMC3741632

[B54] Imaizumi Y. Okano H. (2014). Modeling human neurological disorders with induced pluripotent stem cells. *J. Neurochem.* 129 388–399 10.1111/jnc.12625 24286589

[B55] Itzhaki I. Maizels L. Huber I. Zwi-Dantsis L. Caspi O. Winterstern A. (2011). Modelling the long QT syndrome with induced pluripotent stem cells. *Nature* 471 225–229 10.1038/nature09747 21240260

[B56] James D. Levine A. J. Besser D. Hemmati-Brivanlou A. (2005). TGFbeta/activin/nodal signaling is necessary for the maintenance of pluripotency in human embryonic stem cells. *Development* 132 1273–1282 10.1242/dev.01706 15703277

[B57] Jang M. Lee S. T. Kim J. W. Yang J. H. Yoon J. K. Park J. C. (2013). A feeder-free, defined three-dimensional polyethylene glycol-based extracellular matrix niche for culture of human embryonic stem cells. *Biomaterials* 34 3571–3580 10.1016/j.biomaterials.2013.01.073 23422594

[B58] Jin S. Yao H. Weber J. L. Melkoumian Z. K. Ye K. (2012). A synthetic, xeno-free peptide surface for expansion and directed differentiation of human induced pluripotent stem cells. *PLoS ONE* 7:e50880 10.1371/journal.pone.0050880 PMC3511414 23226418

[B59] Joannides A. Fiore-Heriche C. Westmore K. Caldwell M. Compston A. Allen N. (2006). Automated mechanical passaging: a novel and efficient method for human embryonic stem cell expansion. *Stem Cells* 24 230–235 10.1634/stemcells.2005-0243 16510428

[B60] Khetan S. Burdick J. A. (2010). Patterning network structure to spatially control cellular remodeling and stem cell fate within 3-dimensional hydrogels. *Biomaterials* 31 8228–8234 10.1016/j.biomaterials.2010.07.035 20674004

[B61] Kilian K. A. Bugarija B. Lahn B. T. Mrksich M. (2010). Geometric cues for directing the differentiation of mesenchymal stem cells. *Proc. Natl. Acad. Sci. U.S.A.* 107 4872–4877 10.1073/pnas.0903269107 20194780 PMC2841932

[B62] Kilian K. A. Mrksich M. (2012). Directing stem cell fate by controlling the affinity and density of ligand-receptor interactions at the biomaterials interface. *Angew. Chem. Int. Ed. Engl.* 51 4891–4895 10.1002/anie.201108746 22505230 PMC3754806

[B63] Kingham E. White K. Gadegaard N. Dalby M. J. Oreffo R. O. (2013). Nanotopographical cues augment mesenchymal differentiation of human embryonic stem cells. *Small* 9 2140–2151 10.1002/smll.201202340 23362187

[B64] Klim J. R. Li L. Wrighton P. J. Piekarczyk M. S. Kiessling L. L. (2010). A defined glycosaminoglycan-binding substratum for human pluripotent stem cells. *Nat. Methods* 7 989–994 10.1038/nmeth.1532 21076418 PMC2994976

[B65] Kong Y. P. Tu C. H. Donovan P. J. Yee A. F. (2013). Expression of Oct4 in human embryonic stem cells is dependent on nanotopographical configuration. *Acta Biomater.* 9 6369–6380 10.1016/j.actbio.2013.01.036 23391989

[B66] Kuhl S. J. Kuhl M. (2013). On the role of Wnt/beta-catenin signaling in stem cells. *Biochim. Biophys. Acta* 1830 2297–2306 10.1016/j.bbagen.2012.08.010 22986148

[B67] Kuhlmann T. Miron V. Cui Q. Wegner C. Antel J. Bruck W. (2008). Differentiation block of oligodendroglial progenitor cells as a cause for remyelination failure in chronic multiple sclerosis. *Brain* 131(Pt 7), 1749–1758 10.1093/brain/awn096 18515322

[B68] Lee G. N. Ramirez C. Kim H. Zeltner N. Liu B. Radu C. (2012). Large-scale screening using familial dysautonomia induced pluripotent stem cells identifies compounds that rescue IKBKAP expression. *Nat. Biotechnol.* 30 1244–1248 10.1038/nbt.2435 23159879 PMC3711177

[B69] Lee G. Papapetrou E. P. Kim H. Chambers S. M. Tomishima M. J. Fasano C. A. (2009). Modelling pathogenesis and treatment of familial dysautonomia using patient-specific iPSCs. *Nature* 461 402–406 10.1038/nature08320 19693009 PMC2784695

[B70] Li L. Wang B. H. Wang S. Moalim-Nour L. Mohib K. Lohnes D. (2010). Individual cell movement, asymmetric colony expansion, rho-associated kinase, and E-cadherin impact the clonogenicity of human embryonic stem cells. *Biophys. J.* 98 2442–2451 10.1016/j.bpj.2010.02.029 20513387 PMC2877320

[B71] Li L. Bennett S. A. Wang L. (2012). Role of E-cadherin and other cell adhesion molecules in survival and differentiation of human pluripotent stem cells. *Cell. Adh. Migr.* 6 59–70 10.4161/cam.19583 22647941 PMC3364139

[B72] Liu S. Q. Tay R. Khan M. Ee P. L. R. Hedrick J. L. Yang Y. Y. (2010). Synthetic hydrogels for controlled stem cell differentiation. *Soft Matter* 6 67–81 10.1039/b916705f

[B73] Li Z. Rana T. M. (2012). A kinase inhibitor screen identifies small-molecule enhancers of reprogramming and iPS cell generation. *Nat. Commun.* 3 1085 10.1038/ncomms2059 PMC3658009 23011139

[B74] Lutolf M. P. (2009). Biomaterials: spotlight on hydrogels. *Nat. Mater.* 8 451–453 10.1038/nmat2458 19458644

[B75] Lutolf M. P. Blau H. M. (2009). Artificial stem cell niches. *Adv. Mater.* 21 3255–3268 10.1002/adma.200802582 20882496 PMC3099745

[B76] Lutolf M. P. Gilbert P. M. Blau H. M. (2009). Designing materials to direct stem-cell fate. *Nature* 462 433–441 10.1038/nature08602 19940913 PMC2908011

[B77] Makhortova N. R. Hayhurst M. Cerqueira A. Sinor-Anderson A. D. Zhao W. N. Heiser P. W. (2011). A screen for regulators of survival of motor neuron protein levels. *Nat. Chem. Biol.* 7 544–552 10.1038/nchembio.595 21685895 PMC3236614

[B78] Mathieu J. Zhang Z. Nelson A. Lamba D. A. Reh T. A. Ware C. (2013). Hypoxia induces re-entry of committed cells into pluripotency. *Stem Cells* 31 1737–1748 10.1002/stem.1446 23765801 PMC3921075

[B79] Maury Y. Gauthier M. Peschanski M. Martinat C. (2012). Human pluripotent stem cells for disease modelling and drug screening. *Bioessays* 34 61–71 10.1002/bies.201100071 22038777

[B80] McBeath R. Pirone D. M. Nelson C. M. Bhadriraju K. Chen C. S. (2004). Cell shape, cytoskeletal tension, and RhoA regulate stem cell lineage commitment. *Dev. Cell* 6 483–495 10.1016/S1534-5807(04)00075-9 15068789

[B81] Mei Y. Saha K. Bogatyrev S. R. Yang J. Hook A. L. Kalcioglu Z. I. (2010). Combinatorial development of biomaterials for clonal growth of human pluripotent stem cells. *Nat. Mater.* 9 768–778 10.1038/nmat2812 20729850 PMC3388774

[B82] Meng Y. Eshghi S. Li Y. J. Schmidt R. Schaffer D. V. Healy K. E. (2010). Characterization of integrin engagement during defined human embryonic stem cell culture. *FASEB J.* 24 1056–1065 10.1096/fj.08-126821 19933311 PMC2845424

[B83] Metters A. Hubbell J. (2005). Network formation and degradation behavior of hydrogels formed by Michael-type addition reactions. *Biomacromolecules* 6 290–301 10.1021/bm049607o 15638532

[B84] Miller J. D. Ganat Y. M. Kishinevsky S. Bowman R. L. Liu B. Tu E. Y. (2013). Human iPSC-based modeling of late-onset disease via progerin-induced aging. *Cell Stem Cell* 13 691–705 10.1016/j.stem.2013.11.006 24315443 PMC4153390

[B85] Mitalipova M. M. Rao R. R. Hoyer D. M. Johnson J. A. Meisner L. F. Jones K. L. (2005). Preserving the genetic integrity of human embryonic stem cells. *Nat. Biotechnol.* 23 19–20 10.1038/nbt0105-19 15637610

[B86] Moore H. (2006). The medium is the message. *Nat. Biotechnol.* 24 160–161 10.1038/nbt0206-160 16465158

[B87] Mrksich M. Whitesides G. M. (1995). Patterning self-assembled monolayers using microcontact printing–a new technology for biosensors. *Trends Biotechnol.* 13 228–235 10.1016/S0167-7799(00)88950-7

[B88] Musah S. Morin S. A. Wrighton P. J. Zwick D. B. Jin S. Kiessling L. L. (2012). Glycosaminoglycan-binding hydrogels enable mechanical control of human pluripotent stem cell self-renewal. *ACS Nano* 6 10168–10177 10.1021/nn3039148 23005914 PMC3509190

[B89] Nagakawa Y. Kasuya K. Bunso K. Hosokawa Y. Kuwabara H. Nakagima T. (2014). Usefulness of multi-3-dimensional computed tomograms fused with multiplanar reconstruction images and peroral cholangioscopy findings in hilar cholangiocarcinoma. *J. Hepatobiliary Pancreat. Sci.* 21 256–262 10.1002/jhbp.85 24520072

[B90] Narkilahti S. Rajala K. Pihlajamaki H. Suuronen R. Hovatta O. Skottman H. (2007). Monitoring and analysis of dynamic growth of human embryonic stem cells: comparison of automated instrumentation and conventional culturing methods. *Biomed. Eng. Online* 6:11 10.1186/1475-925X-6-11 PMC1854905 17428350

[B91] Niakan K. K. Eggan K. (2013). Analysis of human embryos from zygote to blastocyst reveals distinct gene expression patterns relative to the mouse. *Dev. Biol.* 375 54–64 10.1016/j.ydbio.2012.12.008 23261930

[B92] Nichols J. Smith A. (2009). Naive and primed pluripotent states. *Cell Stem Cell* 4 487–492 10.1016/j.stem.2009.05.015 19497275

[B93] Niessen C. M. Leckband D. Yap A. S. (2011). Tissue organization by cadherin adhesion molecules: dynamic molecular and cellular mechanisms of morphogenetic regulation. *Physiol. Rev.* 91 691–731 10.1152/physrev.00004.2010 21527735 PMC3556819

[B94] Ohgushi M. Matsumura M. Eiraku M. Murakami K. Aramaki T. Nishiyama A. (2010). Molecular pathway and cell state responsible for dissociation-induced apoptosis in human pluripotent stem cells. *Cell Stem Cell* 7 225–239 10.1016/j.stem.2010.06.018 20682448

[B95] Pan F. Zhang M. Wu G. Lai Y. Greber B. Scholer H. R. (2013). Topographic effect on human induced pluripotent stem cells differentiation towards neuronal lineage. *Biomaterials* 34 8131–8139 10.1016/j.biomaterials.2013.07.025 23891397

[B96] Parrinello S. Samper E. Krtolica A. Goldstein J. Melov S. Campisi J. (2003). Oxygen sensitivity severely limits the replicative lifespan of murine fibroblasts. *Nat. Cell Biol.* 5 741–747 10.1038/ncb1024 12855956 PMC4940195

[B97] Peerani R. Rao B. M. Bauwens C. Yin T. Wood G. A. Nagy A. (2007). Niche-mediated control of human embryonic stem cell self-renewal and differentiation. *EMBO J.* 26 4744–4755 10.1038/sj.emboj.7601896 17948051 PMC2080799

[B98] Pryzhkova M. V. Harris G. M. Ma S. G. Jabbarzadeh E. (2013). Patterning pluripotent stem cells at a single cell level. *J. Biomater. Tissue Eng.* 3 461–471 10.1166/jbt.2013.1106 PMC6101254 30135745

[B99] Robinton D. A. Daley G. Q. (2012). The promise of induced pluripotent stem cells in research and therapy. *Nature* 481 295–305 10.1038/nature10761 22258608 PMC3652331

[B100] Rodin S. Antonsson L. Niaudet C. Simonson O. E. Salmela E. Hansson E. M. (2014). Clonal culturing of human embryonic stem cells on laminin-521/E-cadherin matrix in defined and xeno-free environment. *Nat. Commun.* 5:3195 10.1038/ncomms4195 24463987

[B101] Rowland T. J. Miller L. M. Blaschke A. J. Doss E. L. Bonham A. J. Hikita S. T. (2010). Roles of integrins in human induced pluripotent stem cell growth on matrigel and vitronectin. *Stem Cells Dev.* 19 1231–1240 10.1089/scd.2009.0328 19811096

[B102] Ruiz S. A. Chen C. S. (2007). Microcontact printing: a tool to pattern. *Soft Matter* 3 168–177 10.1039/b613349e 32680260

[B103] Saha K. Mei Y. Reisterer C. M. Pyzocha N. K. Yang J. Muffat J. (2011). Surface-engineered substrates for improved human pluripotent stem cell culture under fully defined conditions. *Proc. Natl. Acad. Sci. U.S.A.* 108 18714–18719 10.1073/pnas.1114854108 22065768 PMC3219112

[B104] Salasznyk R. M. Klees R. F. Williams W. A. Boskey A. Plopper G. E. (2007). Focal adhesion kinase signaling pathways regulate the osteogenic differentiation of human mesenchymal stem cells. *Exp. Cell Res.* 313 22–37 10.1016/j.yexcr.2006.09.013 17081517 PMC1780174

[B105] Sands R. W. Mooney D. J. (2007). Polymers to direct cell fate by controlling the microenvironment. *Curr. Opin. Biotechnol.* 18 448–453 10.1016/j.copbio.2007.10.004 18024105 PMC2262952

[B106] Serra M. Brito C. Correia C. Alves P. M. (2012). Process engineering of human pluripotent stem cells for clinical application. *Trends Biotechnol.* 30 350–359 10.1016/j.tibtech.2012.03.003 22541338

[B107] Sherman S. P. Alva J. A. Thakore-Shah K. Pyle A. D. (2011). Human pluripotent stem cells: the development of high-content screening strategies. *Methods Mol. Biol.* 767 283–295 10.1007/978-1-61779-201-4_21 21822883

[B108] Singh S. Carpenter A. E. Genovesio A. (2014). Increasing the content of high-content screening: an overview. *J. Biomol. Screen.* 19 640–650 10.1177/1087057114528537 24710339 PMC4230961

[B109] Siti-Ismail N. Bishop A. E. Polak J. M. Mantalaris A. (2008). The benefit of human embryonic stem cell encapsulation for prolonged feeder-free maintenance. *Biomaterials* 29 3946–3952 10.1016/j.biomaterials.2008.04.027 18639332

[B110] Smith A. (2006). A glossary for stem-cell biology. *Nature* 441 1060–1060 10.1038/nature04954

[B111] Stevens M. M. George J. H. (2005). Exploring and engineering the cell surface interface. *Science* 310 1135–1138 10.1126/science.1106587 16293749

[B112] Sun Y. Chen C. S. Fu J. (2012a). Forcing stem cells to behave: a biophysical perspective of the cellular microenvironment. *Annu. Rev. Biophys.* 41 519–542 10.1146/annurev-biophys-042910-155306 22404680 PMC4123632

[B113] Sun Y. Villa-Diaz L. G. Lam R. H. Chen W. Krebsbach P. H. Fu J. (2012b). Mechanics regulates fate decisions of human embryonic stem cells. *PLoS ONE* 7:e37178 10.1371/journal.pone.0037178 PMC3353896 22615930

[B114] Sun Y. Fu J. (2013). Mechanobiology: a new frontier for human pluripotent stem cells. *Integr. Biol. (Camb.)* 5 450–457 10.1039/c2ib20256e 23337973 PMC4116275

[B115] Swinney D. C. Anthony J. (2011). How were new medicines discovered? *Nat. Rev. Drug Discov.* 10 507–519 10.1038/nrd3480 21701501

[B116] Tannenbaum S. E. Turetsky T. T. Singer O. Aizenman E. Kirshberg S. Ilouz N. (2012). Derivation of xeno-free and GMP-grade human embryonic stem cells–platforms for future clinical applications. *PLoS ONE* 7:e35325 10.1371/journal.pone.0035325 PMC3380026 22745653

[B117] Teixeira A. I. Abrams G. A. Bertics P. J. Murphy C. J. Nealey P. F. (2003). Epithelial contact guidance on well-defined micro- and nanostructured substrates. *J. Cell Sci.* 116(Pt 10), 1881–1892 10.1242/jcs.00383 12692189 PMC1885893

[B118] Terstegge S. Laufenberg I. Pochert J. Schenk S. Itskovitz-Eldor J. Endl E. (2007). Automated maintenance of embryonic stem cell cultures. *Biotechnol. Bioeng.* 96 195–201 10.1002/bit.21061 16960892

[B119] Tesar P. J. Chenoweth J. G. Brook F. A. Davies T. J. Evans E. P. Mack D. L. (2007). New cell lines from mouse epiblast share defining features with human embryonic stem cells. *Nature* 448 196–199 10.1038/nature05972 17597760

[B120] Thomas R. J. Anderson D. Chandra A. Smith N. M. Young L. E. Williams D. (2009). Automated, scalable culture of human embryonic stem cells in feeder-free conditions. *Biotechnol. Bioeng.* 102 1636–1644 10.1002/bit.22187 19062183

[B121] Thomson J. A. Itskovitz-Eldor J. Shapiro S. S. Waknitz M. A. Swiergiel J. J. Marshall V. S. (1998). Embryonic stem cell lines derived from human blastocysts. *Science* 282 1145–1147 10.1126/science.282.5391.1145 9804556

[B122] Trappmann B. Gautrot J. E. Connelly J. T. Strange D. G. Li Y. Oyen M. L. (2012). Extracellular-matrix tethering regulates stem-cell fate. *Nat. Mater.* 11 642–649 10.1038/nmat3339 22635042

[B123] Treiser M. D. Yang E. H. Gordonov S. Cohen D. M. Androulakis I. P. Kohn J. (2010). Cytoskeleton-based forecasting of stem cell lineage fates. *Proc. Natl. Acad. Sci. U.S.A.* 107 610–615 10.1073/pnas.0909597107 20080726 PMC2818905

[B124] Unadkat H. V. Hulsman M. Cornelissen K. Papenburg B. J. Truckenmuller R. K. Carpenter A. E. (2011). An algorithm-based topographical biomaterials library to instruct cell fate. *Proc. Natl. Acad. Sci. U.S.A.* 108 16565–16570 10.1073/pnas.1109861108 21949368 PMC3189082

[B125] Vallier L. Alexander M. Pedersen R. A. (2005). Activin/Nodal and FGF pathways cooperate to maintain pluripotency of human embryonic stem cells. *J. Cell Sci.* 118(Pt 19), 4495–4509 10.1242/jcs.02553 16179608

[B126] Varelas X. Sakuma R. Samavarchi-Tehrani P. Peerani R. Rao B. M. Dembowy J. (2008). TAZ controls Smad nucleocytoplasmic shuttling and regulates human embryonic stem-cell self-renewal. *Nat. Cell Biol.* 10 837–848 10.1038/ncb1748 18568018

[B127] Vega S. L. Liu E. Patel P. J. Kulesa A. B. Carlson A. L. Ma Y. (2012). High-content imaging-based screening of microenvironment-induced changes to stem cells. *J. Biomol. Screen.* 17 1151–1162 10.1177/1087057112453853 22811477 PMC5944335

[B128] Veraitch F. S. Scott R. Wong J. W. Lye G. J. Mason C. (2008). The impact of manual processing on the expansion and directed differentiation of embryonic stem cells. *Biotechnol. Bioeng.* 99 1216–1229 10.1002/bit.21673 17929326

[B129] Viswanathan P. Chirasatitsin S. Ngamkham K. Engler A. J. Battaglia G. (2012). Cell instructive microporous scaffolds through interface engineering. *J. Am. Chem. Soc.* 134 20103–20109 10.1021/ja308523f 23163574 PMC3556732

[B130] Vogel V. Sheetz M. (2006). Local force and geometry sensing regulate cell functions. *Nat. Rev. Mol. Cell Biol.* 7 265–275 10.1038/nrm1890 16607289

[B131] Ware C. B. Nelson A. M. Mecham B. Hesson J. Zhou W. Jonlin E. C. (2014). Derivation of naive human embryonic stem cells. *Proc. Natl. Acad. Sci. U.S.A.* 111 4484–4489 10.1073/pnas.1319738111 24623855 PMC3970494

[B132] Watanabe K. Ueno M. Kamiya D. Nishiyama A. Matsumura M. Wataya T. (2007). A ROCK inhibitor permits survival of dissociated human embryonic stem cells. *Nat. Biotechnol.* 25 681–686 10.1038/nbt1310 17529971

[B133] Wilding J. L. Bodmer W. F. (2014). Cancer cell lines for drug discovery and development. *Cancer Res.* 74 2377–2384 10.1158/0008-5472.CAN-13-2971 24717177

[B134] Xu X. H. Zhong Z. (2013). Disease modeling and drug screening for neurological diseases using human induced pluripotent stem cells. *Acta Pharmacol. Sin.* 34 755–764 10.1038/aps.2013.63 23685955 PMC3674515

[B135] Xu X. Lei Y. Luo J. Wang J. Zhang S. Yang X. J. (2013). Prevention of beta-amyloid induced toxicity in human iPS cell-derived neurons by inhibition of cyclin-dependent kinases and associated cell cycle events. *Stem Cell Res.* 10 213–227 10.1016/j.scr.2012.11.005 23305945

[B136] Xu Y. Zhu X. Hahm H. S. Wei W. Hao E. Hayek A. (2010). Revealing a core signaling regulatory mechanism for pluripotent stem cell survival and self-renewal by small molecules. *Proc. Natl. Acad. Sci. U.S.A.* 107 8129–8134 10.1073/pnas.1002024107 20406903 PMC2889586

[B137] Yagi T. Ito D. Okada Y. Akamatsu W. Nihei Y. Yoshizaki T. (2011). Modeling familial Alzheimer’s disease with induced pluripotent stem cells. *Hum. Mol. Genet.* 20 4530–4539 10.1093/hmg/ddr394 21900357

[B138] Yahata N. Asai M. Kitaoka S. Takahashi K. Asaka I. Hioki H. (2011). Anti-Abeta drug screening platform using human iPS cell-derived neurons for the treatment of Alzheimer’s disease. *PLoS ONE* 6:e25788 10.1371/journal.pone.0025788 PMC3184175 21984949

[B139] Yang C. S. Lopez C. G. Rana T. M. (2011). Discovery of nonsteroidal anti-inflammatory drug and anticancer drug enhancing reprogramming and induced pluripotent stem cell generation. *Stem Cells* 29 1528–1536 10.1002/stem.717 21898684 PMC3419601

[B140] Yang Y. M. Gupta S. K. Kim K. J. Powers B. E. Cerqueira A. Wainger B. J. (2013). A small molecule screen in stem-cell-derived motor neurons identifies a kinase inhibitor as a candidate therapeutic for ALS. *Cell Stem Cell* 12 713–726 10.1016/j.stem.2013.04.003 23602540 PMC3707511

[B141] Yusa K. Rashid S. T. Strick-Marchand H. Varela I. Liu P. Q. Paschon D. E. (2011). Targeted gene correction of alpha1-antitrypsin deficiency in induced pluripotent stem cells. *Nature* 478 391–394 10.1038/nature10424 21993621 PMC3198846

[B142] Zachar V. Prasad S. M. Weli S. C. Gabrielsen A. Petersen K. Petersen M. B. (2010). The effect of human embryonic stem cells (hESCs) long-term normoxic and hypoxic cultures on the maintenance of pluripotency. *In Vitro Cell Dev. Biol. Anim.* 46 276–283 10.1007/s11626-010-9305-3 20177991

[B143] Zhang D. Pekkanen-Mattila M. Shahsavani M. Falk A. Teixeira A. I. Herland A. (2014). A 3D Alzheimer’s disease culture model and the induction of P21-activated kinase mediated sensing in iPSC derived neurons. *Biomaterials* 35 1420–1428 10.1016/j.biomaterials.2013.11.028 24290439

[B144] Zhang R. Mjoseng H. K. Hoeve M. A. Bauer N. G. Pells S. Besseling R. (2013). A thermoresponsive and chemically defined hydrogel for long-term culture of human embryonic stem cells. *Nat. Commun.* 4:1335 10.1038/ncomms2341 PMC3562446 23299885

[B145] Zhou D. Liu T. Zhou X. Lu G. (2009). Three key variables involved in feeder preparation for the maintenance of human embryonic stem cells. *Cell Biol. Int.* 33 796–800 10.1016/j.cellbi.2009.04.008 19393754

[B146] Zweigerdt R. Olmer R. Singh H. Haverich A. Martin U. (2011). Scalable expansion of human pluripotent stem cells in suspension culture. *Nat. Protoc.* 6 689–700 10.1038/nprot.2011.318 21527925

